# Rigidity of silicone substrates controls cell spreading and stem cell differentiation

**DOI:** 10.1038/srep33411

**Published:** 2016-09-21

**Authors:** Grigory Vertelov, Edgar Gutierrez, Sin-Ae Lee, Edward Ronan, Alex Groisman, Eugene Tkachenko

**Affiliations:** 1 Stemedica Inc., San Diego, CA 92121, USA; 2 Department of Physics, University of California-San Diego, La Jolla, CA 92093, USA; 3 Department of Medicine, University of California-San Diego, La Jolla, CA 92093, USA.

## Abstract

The dependences of spreading and differentiation of stem cells plated on hydrogel and silicone gel substrates on the rigidity and porosity of the substrates have recently been a subject of some controversy. In experiments on human mesenchymal stem cells plated on soft, medium rigidity, and hard silicone gels we show that harder gels are more osteogenic, softer gels are more adipogenic, and cell spreading areas increase with the silicone gel substrate rigidity. The results of our study indicate that substrate rigidity induces some universal cellular responses independently of the porosity or topography of the substrate.

Multiple functions of cells cultured on flat substrates have been shown to depend on the elastic modulus of the substrate, *E*, with the dependence being strongest in a physiological range of soft tissues, corresponding to *E* from 0.1 to 100 kPa. Among those functions are stem cell differentiation, cell spreading, and cell signaling^1^. In the context of differentiation of mesenchymal stem cells (MSCs), substrates with *E* in the ranges of <4 kPa, 8–17 kPa, and >25 kPa, have been classified as soft (adipogenic)^2^,^3^, medium rigidity (myogenic)^1^, and hard (osteogenic)^1^, respectively. In most studies, the soft substrates are hydrogels, and variations in their elastic moduli are usually accompanied by variations in the dry mass and porosity. The paradigm of the effect of substrate rigidity on the cellular functions was challenged by *Trappmann et al*.^4^, who claimed that cell spreading and differentiation on polyacrylamide (PAAm) hydrogel substrates depend not on the elastic moduli of the substrates, but rather on their porosity. The size of the pores in the PAAm substrates changed from 1 μm for soft gels to 0.1 μm for hard gels, affecting the density of adhesion points between the substrate surface and the extracellular matrix (ECM) coating on it^4^. This claim was rebutted by *Wen at al*.^3^, who used hydrogel substrates with different porosities but identical elastic moduli to show that it is the elastic modulus rather than the porosity that is key to the effect of the substrate on cell spreading and differentiation. Both publications agreed, however, that there was no appreciable effect of the substrate rigidity on either cell spreading or differentiation, if the substrate was made of a silicone gel. (Silicone materials normally do not have pores readily detectable under scanning electron microscopy and are structurally uniform down to a scale of at least 100 nm^5^.) This conclusion appears to contradict the findings of several other groups, who reported that when cells are plated on an array of flexible silicone microposts, their spreading and differentiation depend on the rigidity of the substrate^6^,^7^, and that when cell are plated on silicone gels, their differentiation depends on the gel rigidity^8^. To resolve this contradiction, we used commercially available soft, medium, and hard silicone gel substrates with nominal elastic moduli of 0.5, 16, and 64 kPa, respectively, to perform experiments similar to those reported in refs 3 and 4, testing the dependence of differentiation and spreading of MSCs and of spreading of fibroblasts and keratinocytes on the substrate rigidity.

## Results and Discussion

Elastic moduli, *E*, of the silicone gels were measured by assessing the deformation of thin layers of gels under known shear stresses using a previously reported microfluidic technique^9^ (Fig. S1A–C) and a newly developed gel rheometer (Fig. S1D–F). For all three gels and with both measurement techniques, shear strain, *γ*, was a linear function of the shear stress, *τ*, up to the highest tested levels of *γ* (~0.02, ~0.03 and ~0.3 for the 64, 16, and 0.5 kPa gel, respectively). The actual values of *E* obtained from the measurements were consistent with the nominal values of *E* (0.4 and 0.61 kPa for the 0.5 kPa gel,17 and 20 kPa for the 16 kPa gel, and 62 and 65 kPa for the 64 kPa gel; see Supplementary Information for further details). Furthermore, the dependencies of *γ* vs. *τ* for gel layers with thicknesses of 18, 6.1 and 2.4 μm (measured for a gel with a nominal *E* = 2 kPa with a modified version of the microfluidic technique) were nearly indistinguishable from each other and also linear up to the highest tested *γ* of ~0.5 (Fig. S1G). The value of *E* calculated from the measurements (~1.7 kPa) was consistent with the value obtained from measurements on a 1 mm layer of the gel, suggesting that the elastic moduli of the silicone gels are uniform down to a subcellular scale of 2.4 μm. From measurements of shear strain as a function of time after abrupt changes in the shear stress, the relaxation times of the gels were estimated as ~4 s for the 0.5 kPa gel and <1 s for both 16 and 64 kPa gels (Fig. S1H–J). These measurements also indicated that all three gels are true solids that undergo finite deformations in response to shear stress.

**Figure 1:**
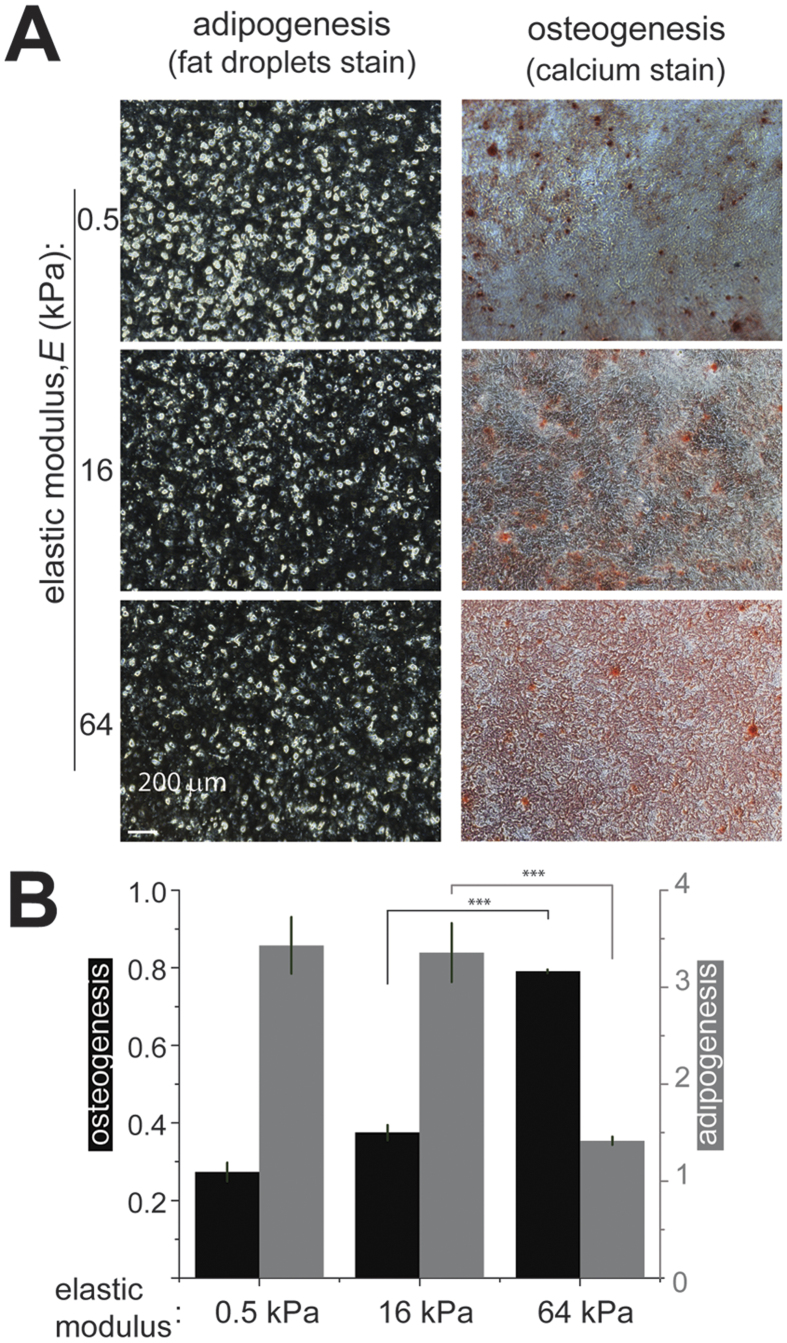
Differentiation of stem cells on substrates of different rigidities. (**A**) Chemically induced adipogenesis (*left column*) and osteogenesis (*right columns*) of hMSCs cultured on silicone gel substrates with different elastic moduli. The *brightness* in the *left column* shows fluorescent staining of adipocytes after 14 days of differentiation. *Red color* in the *right column* corresponds to calcium staining after 14 days of differentiation. (**B**) Chemically induced osteogenesis (*black*) and adipogenesis (*grey*) of human mesenchymal stem cells (hMSCs). *Left* and *right ordinates* indicate the levels of differentiation to osteoblasts and adipocytes, respectively, (n = 3 wells; representative results from 3 independent experiments) normalized to the levels of differentiation of hMSCs plated on plastic surfaces. ***p < 0.01.

In experiments on MSCs, the silicone gel substrates (as well as a plastic substrate used as a control) were coated with collagen I. To study MSCs differentiation, cells were cultured in an adipogenic or an osteogenic medium for 14 days. In an adipogenic medium (Fig. 1A,B), when MSCs were plated on the 64 kPa substrate, their differentiation to adipocytes somewhat increased as compared to a plastic substrate control, and when the MSCs were plated on the 16 kPa and 0.5 kPa substrates, their differentiation to adipocytes increased > 3-fold. In an osteogenic medium (Fig. 1A,B), the differentiation of MSCs to osteoblasts was reduced to ~80% on the 64 kPa substrate as compared with a plastic control and was further reduced to ~36% on the 16 kPa substrate and to ~27% on the 0.5 kPa substrate, with the differences between the three substrates and the control being all significant.

**Figure 2:**
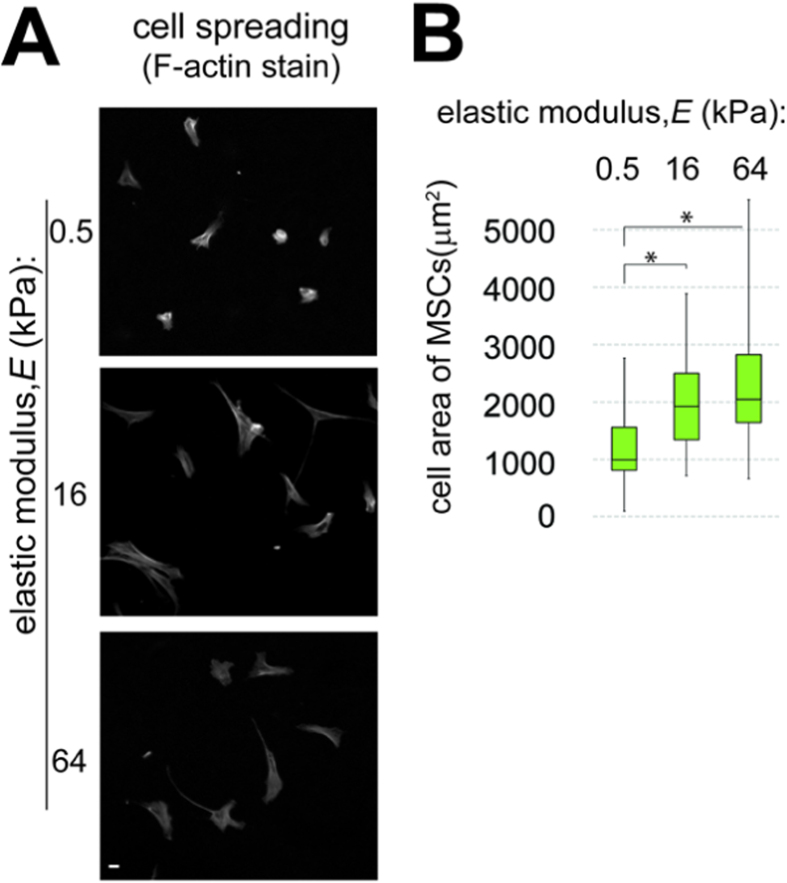
Spreading of stem cells on substrates of different rigidities. (**A**) Representative fluorescence images of hMSCs on silicone substrates with elastic moduli of 0.5, 16, and 64 kPa. The substrates were coated with collagen I and cells were stained with phalloidin to fluorescently label F-actin. (**B**) Spreading areas of hMSCs on silicone substrates with different elastic moduli obtained from the analysis of the fluorescence images. Box corresponds to interquartile range of cell spreading areas; black line indicates median value; whiskers show minimal and maximal values. N = 40 cells for each type of substrates. *statistical significance with p < 0.01.

**Figure 3:**
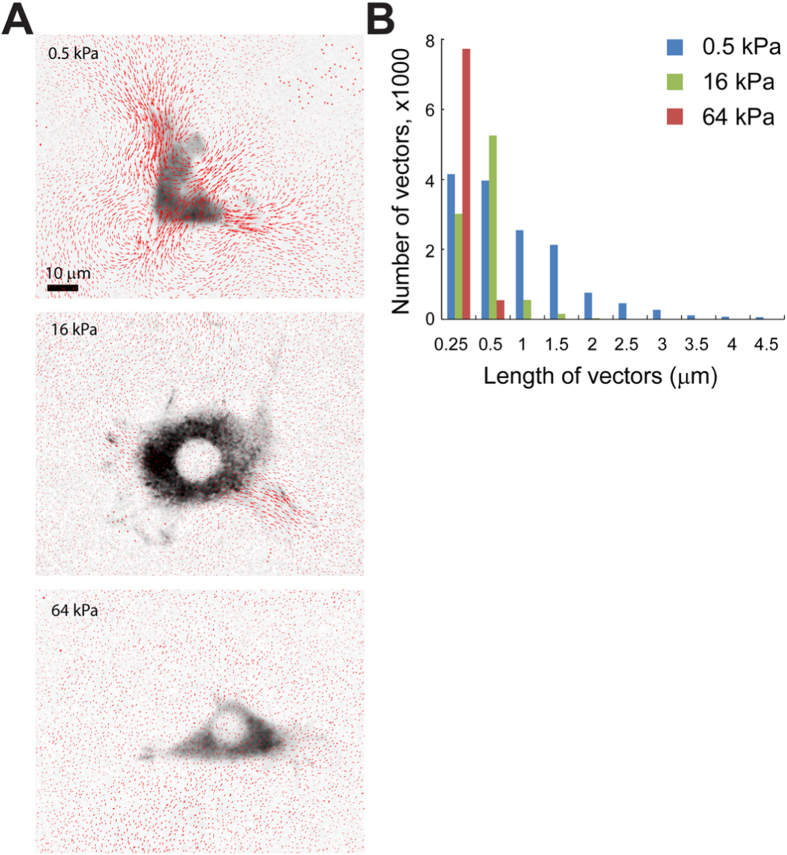
Cell-induced deformations of substrates of different rigidities. (**A**) Inverted greyscale fluorescence images of fibroblasts plated on substrates with *E* = 0.5, 16, and 64 kPa superimposed with vector maps of the surface displacement (*red arrows*). Fibroblasts are expressing paxillin-mCherry. Vector maps are obtained by tracking the displacements of 40 nm red fluorescent beads attached to the substrate surface^3^,^9^. (**B**) Histograms of the lengths of surface displacement vectors for substrates of different rigidities.

In experiments on the spreading of MSCs keratinocytes and fibriblasts, a regular cell culture medium was used and cell spreading areas were assessed 45 minutes after cells were plated. The average spreading areas of MSCs were significantly smaller on the 0.5 kPa silicone gel than on the 16 and 64 kPa gels (Fig. 2A,B). The average areas of primary mouse keratinocytes and mouse embryonic fibroblasts (MEFs) cultured on the silicone gel substrates monotonically increased with the substrate elastic moduli, with differences in the cell areas between the three substrate rigidities being all significant for both cell types (Fig. S2A,B). In agreement with the previous report^10^, we found the phosphorylation level of focal adhesion kinase (FAK) to monotonically increase with the substrate rigidity for both keratinocytes and MEFs (Fig. S2C). Finally, deformations of the silicone gel substrates by traction forces of adherent MEFs were inverse functions of the substrate rigidity and had magnitudes comparable to those reported on hydrogels of similar elastic moduli^3^,^11^ (Fig. 3). Therefore, in all four types of assays, the dependence of the cellular functions on the substrate rigidity was qualitatively the same as for cells cultured on hydrogels and micropost arrays, suggesting that the effects of substrate rigidity on functions of plated cells are similar for all types of deformable substrates. These results demonstrate that substrate rigidity induces some universal cellular responses that are independent of porosity or topography of the substrate.

To explain the discrepancies between our findings and the conclusions of refs 3 and 4 we note that, whereas we plated cells on substrates from all three ranges of rigidity, none of the silicone gel substrates used in refs 3 and 4 was clearly shown to be either soft or of medium rigidity (Fig. S3; Supplementary Discussion). In addition, the surfaces of silicone gel substrates used in our study have amino-reactive groups (Fig. S4), providing covalent binding of ECM proteins similar to the binding of ECM to the surfaces of hydrogels in refs 3 and 4. It is not completely clear, whether the ECM binding to the silicone gel surfaces used in refs 3 and 4 was covalent or passive, and as argued in both papers, cellular responses to the substrate rigidity are expected to depend on the details of binding of ECM to the substrate (see Supplementary Discussion).

## Materials and Methods

### Silicone gel substrates for cell culture

6-well plates with silicone gels on the well bottoms (SoftSubstrates™) were obtained from MuWells (softsubstrates.com, San Diego, CA).

### Measurements of elastic moduli of silicone gels substrates

Elastic moduli of the silicone gels were measured by assessing the deformation of thin layers of gels under known shear stresses (Fig. S4) using a previously reported microfluidic technique^9^ (See Supplementary Methods).

### Assaying density of amine-binding sites on silicone gel substrates

To assess the density of amine-binding sites on the silicone gel substrates Fig. S4), we used fluorescent beads functionalized with amine groups (See Supplementary Methods).

### Stem cell differentiation

Human mesenchymal stem cells (hMSCs) of early passages (P0) were obtained from Stemedica (San Diego, CA). Silicone gels substrates were coated with 1.6 μg/ml solution of collagen I (Advanced Biomatrix, San Diego, CA) in pH 7.4 PBS for 30 min at 37 °C. hMSCs were seeded into the 6-well plates at 600 cells/ cm^2^ in 2 mL of 7.5% BGS (EquaFETAL®, AtlasBIOLOGICALS) hMSC growth media (Stemedica) and grown in humidified oxygen-controlled 37 °C incubator with 5% O_2_ and 5% CO_2_. Cells were allowed to reach ~75% confluence before a differentiation medium (ThermoFisher) was applied to induce either adipogenesis or osteogenesis. After 7 days, the differentiation medium was refreshed, and after 14 days cell were examined to assess their differentiation. Adiposeness was assessed using AdipoRed (ThermoFisher), according to a protocol recommended by the manufacturer with the following modifications: prior to the addition of AdipoRed, all cells from the wells of a 6-well plate were harvested by trypsinization, washed once in pH7.4 PBS, resuspended in 1.2 ml of PBS and transferred into a 96-well plate (200 uL of cell suspension per well); AdipoRed was added to each well of the 96-well plate, incubated for 20 min at RT, and the intensity of staining was measured using a fluorimeter (FLX800, Biotech Instruments Inc). Osteoblasts were stained with Alizarin Red and imaged using Evos FL cell imaging system (Advanced Microscopy Group, Mill Creek, WA), with the level of osteogenesis assessed as previously described^12^. Alternately, osteogenesis was assayed as described in refs 4. Briefly, hMSCs were seeded at a density 2,000 cells/cm^2^, cultured for 1 hour, and the differentiation medium was applied. Cells were assayed for ALP activity after 7 days.

### Cell spreading assay

To measure hMSC spreading areas, hMSCs were plated onto silicone gel substrates in 6-well plates and cultured for 24 hours in 2 mL of 7.5% BGS (EquaFETAL®, AtlasBIOLOGICALS) hMSC growth media (Stemedica) and grown in humidified oxygen-controlled 37 °C incubator with 5% O_2_ and 5% CO_2_. Cells were then fixed with 3.7% formaldehyde in PBS, permeabilized with 0.5% Triton X-100 in PBS at RT for 10 min, and washed three times with PBS. The fixed cells were incubated with phalloidin-conjugated rhodamine (Molecular Probes) for 45 min at RT and washed three times with PBS. Next, cells were photographed under a fluorescence microscope. Mouse primary keratinocytes and mouse embryonic fibroblasts (MEFs) were plated on ~30 μm layers of the 0.5, 16, and 64 kPa silicone gels on #1.5 microscope cover glasses (special order from MuWells), making it possible to measure their spreading areas under fluorescence microscope with improved resolution. Silicone gel surfaces were coated with fibronectin (ThermoFisher) by incubation under a 20 μg/ml solution of fibronectin in pH 7.4 PBS for 30 min at RT. Keratinocytes and MEFs were plated on fibronectin-coated silicone gels and incubated in DMEM supplemented with 1% (v/v) BSA for 45 min at 37 °C, 5% CO_2_. Cells were fixed and stained with phalloidin as described above. Next, the cover glasses were mounted on microscope slides with a mounting solution (ProLong^®^ Gold antifade reagent; Invitrogen) and cells were photographed under a fluorescence microscope. The micrographs were digitally processed and cell spreading areas were quantified using a code in MATLAB (MathWorks, Natick, MA). For each substrate elastic modulus and each cell type, the spreading areas were measured for 75 cells in randomly selected areas of the substrate (Fig. 1D; Fig. S2B).

### Preparation of cell lysates and Western blotting

Keratinocytes and MEFs were plated on fibronectin-coated silicone gel substrates in the 6-well plates and incubated for 45 min at 37 °C, 5% CO2. Whole cell lysates were prepared using modified radioimmune precipitation assay buffer (50 mM Tris, pH 7.5, 150 mM NaCl, 50 mM NaF, 1 mM sodium pyrophosphate, 0.1% sodium deoxycholate, 1% Nonidet P-40, protease inhibitors cocktail and 1% CHAPS). Lysate protein was quantified using the bicinchoninic acid (BCA) method (ThermoFisher Scientific), normalized, and used in Western blots analysis. The primary antibodies for Western blots were against Y^576^FAK (ThermoFisher), FAK (Cell Signaling), and α-tubulin (Sigma) (Fig. S2C).

## Additional Information

**How to cite this article**: Vertelov, G. *et al*. Rigidity of silicone substrates controls cell spreading and stem cell differentiation. *Sci. Rep.* **6**, 33411; doi: 10.1038/srep33411 (2016).

## Supplementary Material

Supplementary Information
